# A Spline‐Based Approach to Smoothly Constrain Hazard Ratios With a View to Apply Treatment Effect Waning

**DOI:** 10.1002/sim.70035

**Published:** 2025-03-09

**Authors:** Angus C. Jennings, Mark J. Rutherford, Paul C. Lambert

**Affiliations:** ^1^ MRC Clinical Trials Unit at UCL, UCL London UK; ^2^ Biostatistics Research Group, Department of Population Health Sciences University of Leicester Leicester UK; ^3^ Norwegian Institute of Public Health, Cancer Registry of Norway Oslo Norway; ^4^ Department of Medical Epidemiology and Biostatistics Karolinska Institutet Stockholm Sweden

**Keywords:** hazard ratios, health technology assessment, splines, survival analysis, treatment efficacy waning

## Abstract

**Objectives:**

To describe and assess, via simulation, a constraint‐based spline approach to implement smooth hazard ratio (HR) waning in time‐to‐event analyses.

**Methods:**

A common consideration when extrapolating survival functions to evaluate the long‐term performance of a novel intervention is scenarios where the beneficial effect of an intervention eventually disappears (treatment effect waning). One approach to relaxing the proportional hazards assumption for a treatment effect is to model it as a function of the timescale, with a spline function offering a flexible approach. We consider the constraint of coefficients of spline variables to 0 during estimation, leading to log‐treatment effects that are constrained to 0 (HR = 1) from a given time‐point: enforcing treatment efficacy waning. An example is reported. Datasets were simulated under a variety of scenarios and analyzed with treatment effect waning assumptions under various modeling choices. Bias in mean survival time difference, given fully observed waning or fully censored waning, was assessed and constrained HR estimates were visualized.

**Results:**

Given full waning, biases were small unless constraints directly contradicted truths. When waning was extrapolated, akin to real‐life practice, biases over observed periods were minimized through the inclusion of a knot at the 95th percentile. The rate at which the HR waned slowed as the upper boundary knot/constraint was placed later, inducing less conservative treatment effect waning assumptions.

**Conclusion:**

An alternative approach to modeling smooth treatment efficacy waning is demonstrated, enabling HR conditioning and marginal RMST calculation in a single framework, along with applications of the method beyond this use.

## 1 Introduction

In order to use estimates of lifetime treatment benefit for health technology assessment (HTA) decision making, extrapolation of effects beyond trial follow‐up is required. With a paucity of long‐term data, there is much debate around assumptions of life‐long treatment benefit. Investigation of treatment effect waning is recommended by the National Institute of Health and Care Excellence (NICE) for use in HTA [1]. Scenarios where the treatment effect stops after a given timepoint or diminishes gradually over time are suggested for consideration.

NICE submissions involving treatment effect waning most commonly come from oncology, specifically immuno‐oncology [2], where treatment stopping rules are frequent, and discussion on how to deal with this is common. Other examples of use are from multiple sclerosis HTAs [3]. To implement waning in a time‐to‐event analysis (often part of partitioned survival or multi‐state model based analyses in HTA [4, 5]) a constant hazard ratio (HR), that is, under proportional hazards (PH) assumptions, may be constrained to 1 from a given point or waned in piecewise constant steps [6] (e.g., half the treatment benefit assumed for 2 years following trial end, a quarter for the 2 years following this and no effect extrapolated from this point to a lifetime horizon).

A recent review of NICE Technology Appraisals by Trigg et al. stated that literature published on methodology to perform this waning is sparse [4]. Perhaps the most common approach used in practice is to implement waning in a post‐estimation step, whereby hazards are predicted for both treatment groups, treatment‐group hazards are replaced with comparator hazards from a decided timepoint, and survival estimates are derived based on numerical integration of these “composite” hazards. Methods by which the effects of waning can be incorporated when fitting the model, as opposed to imposing it as part of a post‐estimation procedure, are less common but might be achieved by including a time‐dependent treatment covariate in a standard parametric model (e.g., a Weibull model) that is a constant value of 1 over the non‐waning period and reaches a value of 0 by the point of desired full waning; the covariate estimate for this term will correspond to the PH effect over the non‐waning period, and any survival/hazard estimates from this model will reflect the chosen waning pattern. This requires full specification of the waning HR shape and a restrictive [7] PH assumption over the non‐waning period.

Previous work has demonstrated the importance of conditioning on key prognostic covariates when applying waning assumptions to the treatment effect HR, with potential for bias in survival difference to result without [8]. Given that HTA decision making is done at a population‐level, it is also important that final estimates are marginal; hence, techniques such as regression standardization can be used to derive marginal survival difference from conditional estimates. This process requires the ability to fully specify the conditional form of adjustments, apply waning constraints, and calculate predicted survival estimates for a range of times for all participants.

We propose a spline‐based method to apply treatment effect waning assumptions to HRs in a time‐to‐event analysis in a smooth, potentially more biologically plausible way without the requirement of cut point/HR multiplier definition nor a restrictive PH assumption over non‐waning periods. It allows conditioning on other key covariates and provides easy predictions for any required covariate patterns in a unified modeling framework and incorporates the effect of waning when fitting the model, as opposed to imposing it as part of a post‐estimation procedure. In the flexible parametric survival model (FPM) framework [9], introduced in a NICE HTA setting in NICE guidelines [10], natural cubic splines are implemented to fully specify the baseline hazard over time, requiring only the definition of spline knots. A similar spline may then be used to model a flexible, time‐varying treatment effect over follow‐up. Appropriate constraints of spline parameters can allow modeling of a HR that will smoothly approach 1, reaching this value by a defined time‐point.

This paper is organized as follows: the new method is introduced along with an example of use and a discussion of R [11]/Stata [12] code in “Methods.” A simulation study, with the primary aim of ascertaining the important modeling decisions required of an analyst and their implications, is then reported in “Simulation Study.” Implications, some other examples of use, and limitations are covered in “Discussion.”

## 2 Methods

### 2.1 Hazard Modeling and Non‐Proportional Hazards

In time‐to‐event analysis of RCTs, it is typical to assume the impact of the treatment effect on the baseline hazard to be multiplicative on the hazard scale and described by a HR that is constant over the period of follow‐up; that is, satisfying the PH assumption (that hazards between groups are proportional over all follow‐up). This means its impact can be summarized by a single number: a convenient property. The PH assumption, however, has faced criticism, and accurate detection of violations has been demonstrated to be difficult [7].

As such, several models have been developed to relax this assumption. One of which is the Royston and Parmar model/FPM, first introduced on the log‐cumulative hazard scale [9], but since extended to the log‐hazard scale [13]. This models the log baseline hazard as a flexible function (a spline, discussed in further detail in the following section) and allows for an equally flexible definition of time‐varying, multiplicative covariate effects. This can be an important tool when a PH assumption is not appropriate or when, in a HTA setting, extrapolation of a constant, protective treatment effect to a lifetime horizon may be deemed too anti‐conservative, given the lacking data to support this.

### 2.2 Conditional Versus Marginal Hazards

A further consideration when modeling hazards is whether, and by what, to condition them on. The marginal hazard at time t gives the population‐level, average instantaneous failure rate of those still in the risk set at t, see Equation (1), where X corresponds to the binary treatment assignment. A conditional hazard gives the hazard rate for a subset of the population with a given set of covariate values: Z=z, see Equation (2). Conditional hazards are generally calculated using adjusted or stratified analyses, whilst marginal hazards might be calculated from an unadjusted analysis or derived from conditional estimates using regression standardization, an example of a covariate‐adjusted marginal treatment effect estimator [14], see Equation (3).

(1) $$h_{M}\left(t|X=x\right)=\lim_{\delta t\to 0}\frac{P\left(t\leq T<t+\delta t\;|\;T\geq t,X=x\right)}{\delta t}$$

(2) $$h\left(t|X=x,Z=z\right)=\lim_{\delta t\to 0}\frac{P\left(t\leq T<t+\delta t\;|\;T\geq t,X=x,Z=z\right)}{\delta t}$$

(3) $${\hat{h}}_{M}\left(t|X=x\right)=\frac{\frac{1}{N}{\text{∑}}_{i=1}^{N}S\left(t|X=x,Z=z_{i}\right)h\left(t|X=x,Z=z_{i}\right)}{\frac{1}{N}{\text{∑}}_{i=1}^{N}S\left(t|X=x,Z=z_{i}\right)}$$

Non‐collapsibility of hazard measures, induced by the requirement of survival up to time t for their estimation at t, means that important differences exist in value and interpretation between marginal and conditional hazards/HRs [15]. Applying treatment effect waning to a marginal HR might not accurately estimate survival differences under individual‐level treatment efficacy waning (of interest given that both waning in drug efficacy and treatment stopping rules happen within individuals) and can overestimate treatment benefit. The full discussion and explanation for this are reserved for previous work [8], but it implies that waning assumptions should be applied to a HR that is conditioned on all prognostic factors possible such that bias might be minimized. For HTA, however, marginal estimates are still of interest given that decision‐making is based on a population level and most economic models are built in this way. As such, conditioning, followed by regression standardization, is an important step for accurate assessment of sensitivity to treatment efficacy waning.

Survival estimates do not suffer from non‐collapsibility, however, regression standardization can be used similarly to derive marginal estimates from conditional ones. This is simply the average of predicted survival probability at time t across the observed covariate distribution, see Equation (4), rather than the weighted average given in Equation (3).

(4) $${\hat{S}}_{M}\left(t|X=x\right)=\frac{1}{N}\sum_{i=1}^{N}S\left(t|X=x,Z=z_{i}\right)$$

The model described herein provides a flexible framework under which conditional HRs can be constrained while allowing predictions such that regression standardization can be used to return to marginal estimates of survival/treatment effect.

### 2.3 The Spline‐Based Method

Splines, or piecewise polynomials, are useful tools to model two‐dimensional relationships with a high degree of flexibility. A brief introduction is provided here, with further details found in references [16].

**FIGURE 1:**
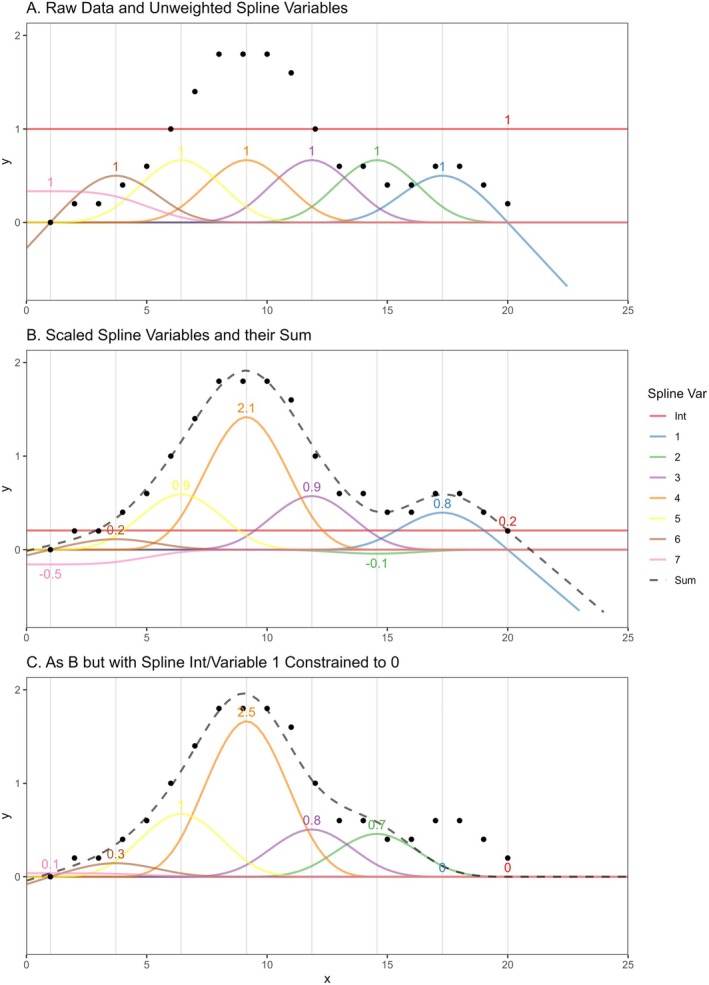
Demonstration of spline variables and their fit to data points with/without constraints. Numbers correspond to estimated/constrained weights applied to spline variables to achieve the Sum value. Gray vertical lines indicate knot (boundary and internal) locations. Spline variable 1 corresponds to the “Forward Extrapolation Spline Variable.”

An *n*th degree spline is formed of k polynomials, each of degree ≤n, for example, a cubic spline is made up of piece‐wise cubic polynomials. Splines are defined such that they are continuous up to r derivatives at each join, with r=0 requiring equality of piecewise functions, whilst r=2 requires equality of second derivatives of piece‐wise functions, at each join. Natural cubic splines are piece‐wise cubic polynomials that are constrained such that they are twice continuously differential at joins and so the second derivative is 0 outside of the spline interval endpoints or “boundary knots”; the spline is linear before the first and after the last knot. This brings benefits when extrapolating using spline functions, as acceleration of the curve at the boundaries is restricted. A spline is fully defined by the k spline “basis” variables and their coefficients that, under linear combinations, form the full spline. Alternatively, the spline variables can be defined by the k+1 knots, or piecewise “joins” (k−1 internal knots and 2 boundary knots). There are several spline variable parameterizations that satisfy the definition of a natural cubic spline, generally defined algorithmically, with one option shown in Figure 1A (for the simple linear regression case). When these spline variables are included as a transformation of another variable (x in Figure 1) in the linear predictor of a model and coefficients estimated for each, an optimum weighted sum is achieved such that the linear combination flexibly fits the data provided, allowing a non‐linear relationship between the outcome and response, see Figure 1B. Whilst natural cubic spline variable parameterisations may differ, predictions based on their sum are equivalent up to knot specification. Splines work in this way across generalized linear and time‐to‐event modeling.

The spline in Figure 1A is made up of 7 spline variables and an intercept. This is the natural cubic spline parameterization employed in the R package “splines2” [17], but with the spline variables defined “backwards,” treating knots in reverse order in the way described by Andersson et al. [18], and equivalent to the definition used in the Stata package “stpm3” [19]. This parameterisation is assumed throughout the definition of this method. Besides the intercept, there is a single spline variable, denoted 1 in Figure 1, that is non‐zero beyond the upper boundary knot (the spline property pivotal for the primary method presented here), herein referred to as the “forward extrapolation spline variable.” This parameterization was chosen so that the full definition of the spline function beyond the upper boundary knot was made up of a minimal number of spline variables.

Constraints can be applied to any parameter in an estimation procedure (simply using the “constraint” command in Stata or via a slightly more complicated/package‐dependent approach in R) to fix its value to a given constant during optimisation, for example whist maximizing the likelihood. This can allow flexible manipulation of final spline characteristics. If both the intercept and forward extrapolation spline variable coefficients were constrained to 0, the final spline and estimated value of y as a function of *x* is forced to be 0 for all x>20, the upper boundary knot, see Figure 1C. Similarly, if the intercept were constrained to 0.1, the total predicted value would be 0.1 for all x>20.

The piecewise nature of spline functions means that constraints can be applied to specific sections of a curve, whilst other sections remain largely unchanged. As the other spline variables are still optimized around these constraints, loss to goodness‐of‐fit within other regions is minimized. In the example provided, the spline value for x<15 is largely unchanged and a smooth approach to 0 that closest fits the observed data given the spline flexibility allowed/constraint enforced is achieved.

Spline functions other than natural cubic splines do not provide the required properties for extrapolation; for example, B‐splines (and, by extension, M‐ and I‐splines that are normalized and integrated B‐splines respectively) that are exactly 0 beyond the upper boundary knot. Extensions for extrapolation, such as periodic B‐splines (and similarly for periodic M/I‐splines), by the nature of their periodicity, could not be constrained in extrapolations without impacting goodness‐of‐fit in the observed data period. This work could be extended to penalized (P) splines, but this is beyond the scope of this paper.

Parameterisations of the natural cubic spline other than that presented here do not allow for the minimal definition of constraints proposed here. For example, in the unaltered splines2 natural cubic spline parameterisation, there are multiple spline variables that are non‐zero beyond the upper boundary knot. As such, a greater number of spline variable coefficients are required to be constrained to 0 to achieve the desired constraint; thus, the overall flexibility of the spline function away from the constrained section is more impacted, increasing the potential for bias in estimates.

Constraining any parameter value artificially enforces a standard error of 0; as far as the model is concerned, this value is absolutely certain and not estimated. This is worthy of note and is discussed further later.

In time‐to‐event analyses, natural cubic splines form the basis of flexible parametric survival models [9, 13]. As described previously, a spline of time (usually log‐transformed) can be used to model a time‐varying (non‐PH), additive log‐HR (multiplicative on the hazard scale) acting on the baseline log‐hazard. If the coefficient for the forward extrapolation *treatment effect* spline variable is constrained to 0, along with the treatment effect spline intercept (usually the PH main effect for treatment), the additive impact of treatment on the log‐hazard scale will be smoothly constrained to 0, and the HR to 1, for all times beyond the chosen upper boundary knot: enforcing HR waning.

A further step is required for this approach to be used to accurately represent treatment effect waning. As both the baseline log‐hazard spline and the additive treatment effect spline are optimized in the face of this constraint, in some cases the optimal baseline spline (commonly directly representing placebo or comparator hazards) might be estimated as a value closer to the hazard experienced by treatment group participants such that equality in treatment group hazards can be achieved. This is not reflective of what would be expected for true treatment effect waning, which would only impact treatment hazards, with comparator hazards completely unchanged. This leads to the 2‐step model procedure outlined below:

1. An unconstrained model is fitted, such that the baseline/placebo hazards can be ascertained without the impact of waning constraints—baseline hazard spline coefficients are saved.
2. A second model is fitted, constraining (a) the baseline spline variable coefficients to those derived in step 1 and (b) the coefficient corresponding to the required treatment spline variables to 0 (namely the PH treatment effect and the forward extrapolation treatment effect spline variable), such that waning is enforced without impacting placebo hazard estimates.

As there is an artificial loss of variance induced by including previously estimated baseline/placebo coefficients as constants, the standard errors derived directly from these models are incorrect. As such, bootstrapping [20] can be used to derive error measures.

This leads to a time‐to‐event model where smooth treatment efficacy waning can be applied to a HR with only the requirement of specification of the hazard spline knot locations (implying the point that the HR = 1 via the upper boundary knot). Survival estimates have been shown to be robust to sensible hazard knot placement [21]. In standard analyses, the number of spline variables (degrees of freedom) will be chosen, and the corresponding number of knots placed at quantiles of the observed data, or the *event* times in the time‐to‐event case. As typically events will be concentrated at earlier times, an extra knot at the 95th percentile, for example, can be included to provide extra flexibility leading up to the point of constraint.

Alternate modeling options exist under this constraints framework, such as enforcing PH beyond the upper boundary knot (by only constraining the forward extrapolation spline variable) or requiring PH over the observed data period followed by waning in the extrapolated period (as briefly outlined in the example below).

An example of model implementation is now demonstrated before being studied closer via simulation.

### 2.4 An Example

To demonstrate, a trial of 619 individuals, comparing Lev+5FU (levamisole and fluorouracil) versus placebo for colon cancer [22], is considered. The maximum follow‐up was 9.06 years, and the maximum death time was 7.64 years. 50.4% were female, with a median age of 62 years. We may wish to extrapolate survival beyond observed follow‐up while assessing sensitivity to the assumption of sustained treatment effect by considering treatment effect waning. This may be done by constraining the HR for the treatment effect to 1 from a given time point. Aligning with the approach suggested in previous work [8], on top of a time‐varying baseline hazard and Lev+5FU treatment effect, all models were adjusted for the covariates: sex, age, whether the colon is obstructed or perforated, tumor adherence to nearby organs, whether there are more than 4 positive lymph nodes, a measure of the extent of local spread, and whether it was a “long” time from surgery (as defined by the study team), to reduce bias in waning estimates. Conditional estimates were based on mean/mode covariate values, and regression standardization was used to derive marginal RMST estimates.

**FIGURE 2:**
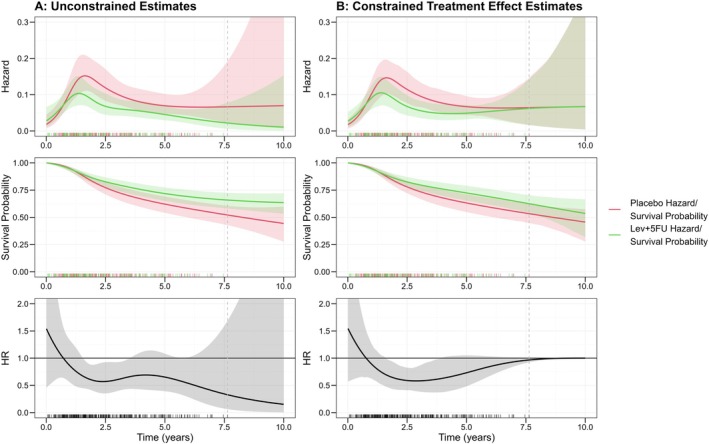
Conditional (on mean/mode covariate values) hazard, survival probability and HR estimates and 95% confidence intervals from unconstrained and constrained HR models, with rug plot of event times. Vertical dashed line indicates last event time. Slight differences in placebo hazards between model A and B are down to small differences in estimated effect of other covariates between the models (e.g., age and sex).

Figure 2A shows unconstrained estimated conditional hazards, survival probability, and HRs based on FPMs with five knots total (including boundary knots) placed at even percentiles of event times (for both the baseline hazard and treatment effect splines). Extrapolation of this non‐PH treatment effect without added constraints would result in a decreasing, increasingly protective treatment effect point estimates over time.

To apply HR waning constraints as described previously, placebo coefficients from the unconstrained model were kept fixed while the required log‐scale treatment effect coefficients were constrained to 0 (the PH treatment effect and forward extrapolation treatment effect spline variable). To allow flexibility to fit the observed data and the constraints simultaneously, one of the internal knots for the treatment effect spline was placed at the 95th percentile and the remaining one at the median event time. The upper boundary knot for the treatment effect spline was moved to 10 years, such that a HR of 1 would be achieved at this time point. The placebo hazard shape was hence unchanged in the final model while the HR was constrained to 1 from 10 years. There were slight differences in the estimated covariate effect of other covariates, due to non‐collapsibility, meaning conditional baseline hazard estimates differed slightly between the models (this might be alleviated by introducing treatment‐covariate interactions, however, this raises further issues of model complexity, among others). The impact of these constraints on estimates is shown in Figure 2B. The 95% confidence intervals for the constrained model were derived using 1000 bootstrap replicates and the percentile method.

The constrained HR can be seen to take a similar shape to the unconstrained HR from years 0–5. From this point, the constrained HR then increases to 1. While the constraint is at 10 years, the constrained HR is adjusted upwards from around 5 years and nears 1 by the end of follow‐up. The constrained HR over the observed follow‐up period is well within 95% confidence intervals of the unconstrained model, which are relatively large due to a smaller number of events nearing the 7‐year timepoint.

30‐year marginal ∆RMST (difference in 30‐year restricted mean survival times = Lev+5FU RMST—Placebo RMST) from these models is now considered, derived via regression standardization [14]. Marginal placebo/Lev+5FU (∆) RMST for the unconstrained model was 9.3/17.2 (7.9). For the model applying constraints, marginal RMSTs were now 9.6/10.9 (1.3), with extrapolated treatment hazards constrained to the more conservative placebo extrapolation. Rather than the treatment effect being extrapolated over the whole 30‐year period (and actually extrapolated as strengthening), it is assumed to be lost shortly following the end of follow‐up; thus, ∆RMST is significantly reduced. While this may be an extreme example given the decreasing treatment effect extrapolated in the unconstrained case, it demonstrates how sensitivity to such assumptions of sustained treatment effect might be assessed in a framework where treatment effect HRs can be conditioned in a simple manner and marginal survival estimates can be derived directly. Small differences in placebo RMST estimates are due to differences in the estimated effect of other covariates modeled, such as age or sex, as mentioned previously.

For a less conservative approach, the upper boundary knot was placed at 15 years instead, resulting in constrained marginal ∆RMST of 1.9 years, increasing the estimated treatment effect by 0.6 years (46%).

To demonstrate a more simplistic, 1‐stage, smooth waning analysis, PH was assumed over the trial follow‐up, with non‐PH waning to 1 imposed after the end of follow‐up; closest replicating the current approach most commonly taken in practice [6]. This was done by shifting the two treatment effect spline boundary knots to the upper event time and 10 years, respectively, and constraining all treatment effect coefficients (including the PH treatment effect) to 0, apart from that corresponding to the spline variable 7 in Figure 1. The constant point estimate over follow‐up was equal to those derived from a standard PH FPM to 3 dp (lower confidence limits were equal to 3dp, whilst upper limits were all equal to 2dp). Marginal ∆RMST for the PH model without waning was 3.3 years, compared to 2.6 years when waning was assumed (and 2.9 when waning instead ended at 15 years). The hazards, survival probabilities, and HRs estimated from these models are shown in Appendix S1. This method is not explored in simulation and hence this is limited to a presentation of a potential alternative method under the presented constraints framework.

These analyses were carried out in R with R and Stata code to reproduce the above example provided on GitHub (https://github.com/angusjennings/spline‐model). R code made use of the survPen package [23], given the superior flexibility in FPM specification afforded by this package, with an altered Newton–Raphson algorithm to allow for constraints. Different packages would require different modeling approaches to constrain parameter estimates (e.g., using the “fixed” or “fixedpars” options in “flexsurvreg” [24] or “mle” (in base R “stats4”) respectively). Stata code made use of the more standard “constraint” command.

### 2.5 Simulation Study

This simulation study is reported in line with the ADEMP (Aims, Data‐generating mechanisms, Methods, Estimands, Performance measures) standard [25], which provides a standard approach by which simulation studies should be planned, analyzed, and reported, with a set of suggested, coherent terminology to facilitate accurate description of the work presented.

#### 2.5.1 Aims

(1) to demonstrate the outlined method given full follow‐up, including waning, and highlight cases where bias (in hazards and RMST) may exist. In practice, full data on waning would rarely exist; hence we also define aim (2) to demonstrate the behavior of extrapolated waning when given no data on effect diminution, identifying the shape of estimated HR waning and corresponding RMST differences under various modeling choices, along with any biases incurred over the observed period. The latter is important for analysis/decision‐making teams to know which modeling choices induce a more or less conservative waning assumption and demonstrate how bias over the observed period might be eliminated.

#### 2.5.2 Data Generating Mechanism

Three hundred datasets (*n*_sim_) each with 600 or 1000 observations (*n*_obs_) were created. For each “participant,” a binomial (p=0.5) treatment assignment and a standard normal frailty measure, $z_{u}$, were generated. Survival times were generated [26, 27] based on covariate values and under several baseline hazard structures (varying shape and level), described in Table 1. The true treatment effect for simulation was a constant, protective level (HR = 0.5) for the first 3 years, before disappearing instantly at this point or smoothly waning (based on a transformed sine curve) to no effect (HR = 1) by 5 or 10 years. Frailty was assumed to act multiplicatively on all hazards, with a HR of 1.5.

**TABLE 1:** Data generating mechanism scenarios.

Observations (*n*_obs_)	BL hazard	Treatment waning
600	Low Exponential (λ=0.15)	Instant (at 3 years)
1000	High Exponential (λ=0.3)	Quick (3–5 years)*
	Low Weibull (λ=0.3, γ=0.5)	Steady (3–10 years)*
	High Weibull (λ=0.7, γ=0.5)	

*Note:* Exponential hazard structure corresponds to a constant baseline hazard (value λ) whilst the chosen Weibull hazard structures correspond to a decreasing baseline hazard. Full list of 24 simulation scenarios derived in a factorial manner (all 24 used in Aim 1, only 8 with instant waning used in Aim 2).

Abbreviation: BL, baseline.

* Quick and Steady waning DGMs are only relevant for Aim 1 models. For Aim 2, all data was censored at 3 years.

The sample size (*n*_obs_) of 600 was chosen to approximate the most recent median sample size for oncological studies reported in a previous systematic review of oncological studies [28]. A larger sample size of 1000 was then chosen to replicate larger oncological studies. Hazard parameters were chosen such that true 5‐year survival probabilities for the low and high hazard scenarios were roughly equal to 5‐year overall survival and progression‐free probability (time to biochemical or radiological progression) respectively, for NICE HTA TA903 [29] (roughly 50% for low hazard and 20% for high hazard). The conditional HR of 0.5 was chosen at a midpoint of the corresponding (partially) conditional HRs reported in TA903. Three hundred repeats (*n*_sim_) were deemed sufficient to achieve an adequately low Monte Carlo standard error of bias estimates given computational intensity.

#### 2.5.3 Estimand

Conditional hazard rates/HRs and marginal restricted survival time difference (∆RMST) under conditional HR constraints to 1.

#### 2.5.4 Models

For the first of the two aims, all data was censored at 20 years. FPMs were fitted to each of the 300 datasets using one internal knot for the log‐time baseline hazard spline and with zero constraints on the PH treatment effect and forward extrapolation treatment effect spline variable coefficients, as described previously. To assess the ability of the model to fit to observed waning under different modeling options, the treatment effect was modeled using 3, 4, and 5 internal knots or, equivalently, 4, 5 and 6 degrees of freedom (df). Lower boundary knots were placed at the lowest observed event time. To enact differing constraints, remaining knots were placed at evenly spaced quantiles of the observed (uncensored) event times less than either 5, 10, or 20 years, with the upper boundary knot placed at exactly these values (where the constraint to 1 will be from). Each of these models was further fitted with/without the inclusion of an extra knot for the treatment spline placed at the 95th percentile of observed event times, used to give more flexibility to return to 1 and effectively fit to observed data.

For Aim 2, similar models to the above were fitted but limited to only 3 years of follow‐up (censoring data before any waning can be observed). In this case, all constraints are enacted outside of the observed data, closer reflecting use in practice. As the observed data is much simpler than for Aim 1 (with exactly proportional hazards in the observed period) the treatment effect spline was modeled with 2, 3, and 4 internal knots (3, 4 and 5 df).

See Table 2 for a summary of models fitted, corresponding to a total of 18 for each aim. For Aim 1, all models were fitted to all 24 datasets. For Aim 2, DGMs corresponding to Quick or Steady waning were dropped (leaving 8 datasets) due to the equivalence of DGMs given 3‐year censoring.

**TABLE 2:** Modeling parameter choices.

Degrees of freedom (BL spline/TRT effect spline)	Year of constraint	Extra 95th percentile knot
Aim 1: Censored 20 years		
BL 1/TRT 4	5	None
BL 1/TRT 5	10	Included
BL 1/TRT 6	20	
Aim 2: Censored 3 years		
BL 1/TRT 3	5	None
BL 1/TRT 4	10	Included
BL 1/TRT 5	20	

*Note:* Full list of models fitted derived in a factorial manner. Degrees of Freedom = Number of Internal Knots +1.

Abbreviations: BL, baseline; TRT, treatment.

To further evaluate this method in a real‐life scenario where a more complex censoring pattern would be observed, all Aim 2 models were re‐fitted, including calendar censoring. A random uniform (on 0–1) entry time (in years from the “enrolment opening,” defined as time 0) was generated for each participant. All those still alive at 2 years from the last participant enrolled (for simplicity here taken to be exactly at 1 year from enrolment opening, even if the true simulated last enrolment time may be slightly less than this) were then censored at this date. The impact of this is random uniform censoring of surviving participants between 2 and 3 years of follow‐up, akin to what might be seen in a trial enrolling over a 1‐year period with a 2‐year minimum follow‐up time.

Heterogeneity $z_{u}$ was adjusted for in all models, corresponding to conditional hazards/HRs. Marginal RMST was derived using regression standardization [14], using the conditional survival estimates and sample $z_{u}$ distributions. This choice is covered in the Discussion.

All analyses were carried out in R [11], with survival times generated using simsurv [30] and FPMs analyses using survPen [23] functionality with an altered Newton–Raphson procedure to allow constraints. All code used for the analysis is provided via GitHub (https://github.com/angusjennings/spline‐model).

#### 2.5.5 Performance Measures

For Aim 1, performance was measured using plots of true/“estimated under constraint” hazards/HRs and percent/absolute bias in marginal RMSTs/∆RMST (Treatment RMST—Placebo RMST).

True marginal RMST was calculated using numerical integration of a KM [31] plot fitted to a single dataset size 1,000,000 generated for each of the hazard structures (Table 1).

For Aim 2, as no data on true waning was provided to models, bias was not considered for extrapolated periods. Instead, only bias in 3‐year marginal RMST was considered. The waning induced by the model under the varying model options was considered using plots of true/“estimated under constraint” hazards/HRs and absolute 40‐year marginal RMST estimates.

In all cases, model convergence was also considered to evaluate performance.

This work is deemed to be Phase II research, per the phases of methodological research proposed by Heinze [32], limiting this paper to the demonstration of the new method and a limited range of possible applications. Further, the authors note the absence of published work detailing comparable methods allowing non‐PH main effects, flexible waning, and, perhaps more importantly for accurate HTA [8], covariate adjustment and regression standardization in a single framework. As such, no comparisons to other methods are planned, and this simulation study is limited to the evaluation of the presented method alone. This is noted as a limitation.

## 3 Results

### 3.1 Aim 1: 20‐Year Censored Data

Aim 1 was to assess model performance, given full waning, under a range of scenarios.

**FIGURE 3:**
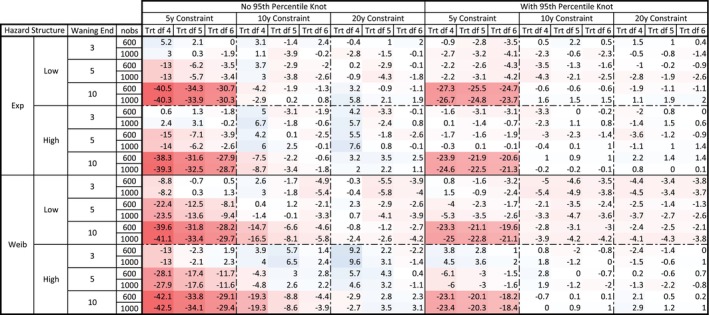
Mean percent bias in 20‐year ∆RMST for all data‐generating mechanisms. df—degrees of freedom (here of the time‐varying treatment effect hazard ratio); Exp—Exponential; Trt—treatment; Weib—Weibull.

The full table of percentage bias in 20‐year ∆RMST (100 × (Estimated ∆RMST—True ∆RMST)/True ∆RMST) is given in Figure 3. Absolute biases are given in Appendix S2. Red values indicate negative bias/conservative estimates whilst blue the converse. All biases in the placebo arm 20‐year RMST estimates were less than 0.09 years.

The primary source of bias when the waning period was *not* censored was constraints that were too early. When constraints (of conditional HR = 1) were placed at 5 years but waning truly ended at 10 years, the RMST difference was biased downwards by up to 42.5% (corresponding to 1.25 years). Without a 95th percentile knot, bias was also elevated with a constraint placed at the true point of waning (e.g., 5 year waning end and 5 year constraint).

Biases were much smaller with the inclusion of the 95th percentile knot (average −0.09 years or −3.6% across all models, versus −0.15 years or −5.8% without). Inclusion of the 95th percentile knot also alleviated bias incurred by placing constraints at the true point of waning. The 95th percentile knot implies a degree of freedom 1 higher than shown in column headings, but comparing models including the 95th percentile knot to models with an extra knot placed at an even percentile maintains a preference for the 95th percentile knot placement.

Generally, increasing the number of parameters for the spline function reduced biases (average −5.5%, −4.5% and −4% for the 4, 5, and 6 df models), this was not always the case.

Excluding cases where the constraint was placed before the true waning ended, average bias across all models was −0.04 years (−1.7%) indicating good model performance.

Model performance was consistent across the baseline hazard structure used (slightly better in terms of percentage bias for the Exponential baseline hazard and high hazard DGMs). Percentage bias was consistent for samples sized 600 and 1000 (mean percent bias for the 1000 case 0.09 units more negative than for 600). Variance in RMST estimates, however, was much greater in the 600‐participant case. Biases generally indicate conservative estimates. A larger number of conservative estimates can be seen for the 3‐year waning end.

### 3.2 Aim 2: 3‐Year Censored Data

Aim 2 was then to (a) consider bias in 3‐year RMST estimates under different models, showing how modeling choices might affect estimates over an observed RCT follow‐up, and (b) demonstrate the HR waning shape enforced by different modeling choices.

**FIGURE 4:**
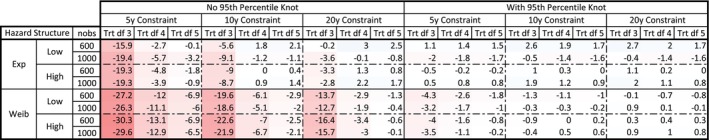
Mean percent bias in 3‐year ∆RMST for data generating mechanisms with waning end at 3 years. df—degrees of freedom (here of the time‐varying treatment effect hazard ratio); Exp—Exponential; Trt—treatment; Weib—Weibull.

Figure 4 shows percentage bias in 3‐year marginal RMST under different models, with data censored at 3 years (prior to any observed waning) in all cases. Only DGMs with waning ending at 3 years were considered (which, given 3‐year follow‐up, is equivalent to both 5/10 year waning end). Absolute biases are given in Appendix S3.

Adding a knot at the 95th percentile of the observed data significantly reduced the average percentage bias (−0.15%, or < 0.001 years, with the 95th percentile knot versus −7.0%, or −0.03 years, without). The maximum mean absolute bias in 3‐year RMST with the 95th percentile knot was 0.024 years. Again, models with this knot generally still outperformed a model with just an added df (where the extra knot will just be evenly spaced across quantiles). Without the 95th percentile knot, biases over observed data periods were larger with an earlier constraint and with a reduced df, as high as 30.3% reduction in the estimated treatment effect if a constraint was placed at 5 years with 3 df for the treatment spline and no 95th percentile knot.

Similar to Aim 1, increasing the treatment effect spline complexity generally decreased biases (average −7.8%, −2.0%, and −0.8% for 3, 4, and 5 df, respectively).

**FIGURE 5:**
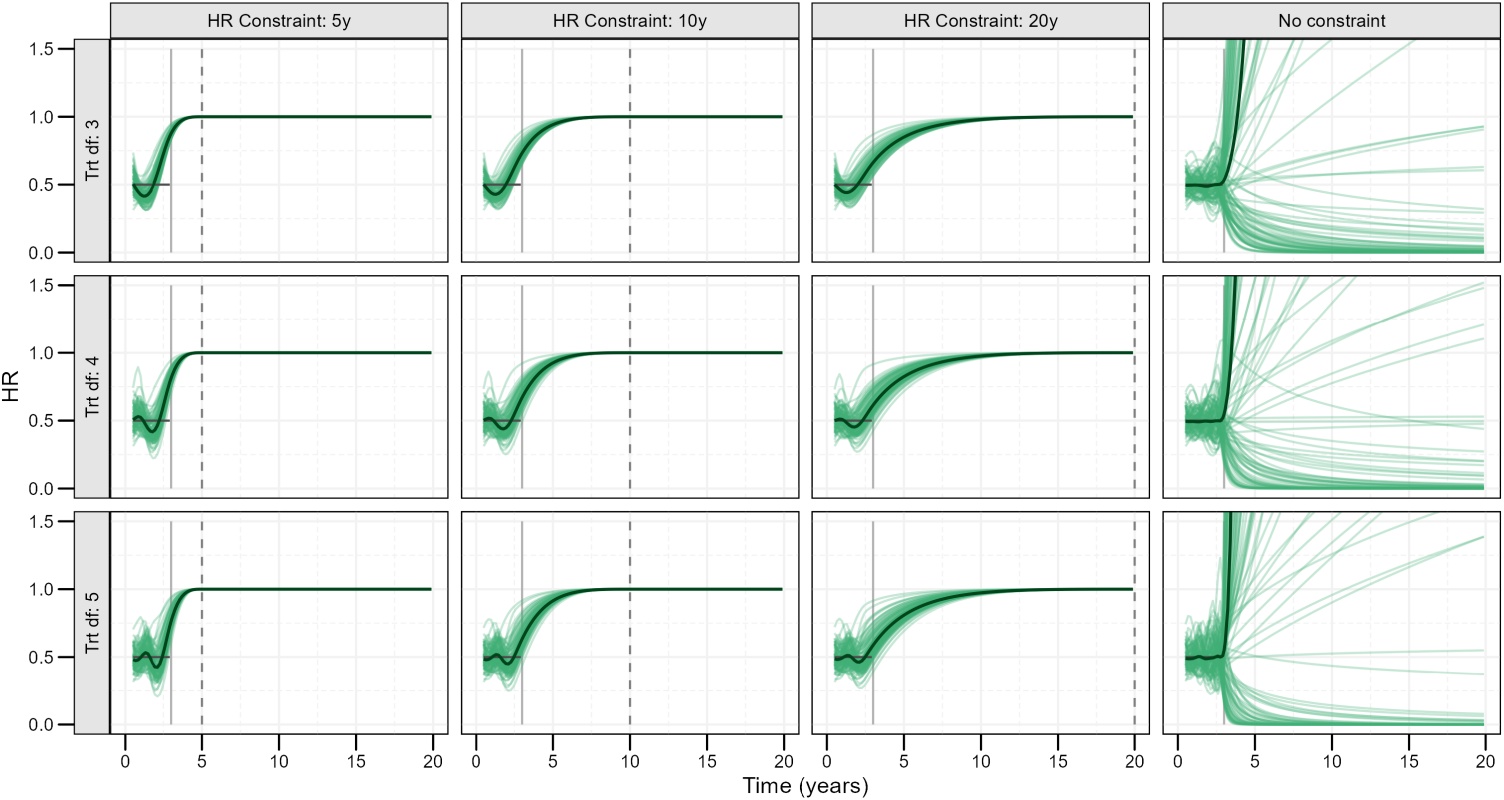
Conditional (on *z* = 0) hazard ratios for the first 100 of 300 simulations under the high exponential hazard data generating mechanism, sized 1000 each, all models including a 95th percentile knot. Darker line corresponds to the average of the 100 simulations (paler lines). The solid vertical line at 3 years indicates point of censoring. The true conditional HR of 0.5 is indicated in gray prior to this point. Dashed vertical lines at 5, 10 and 20 years indicate the point of constraint.

Figure 5 shows the HR estimates from the first 100 simulations based on the high exponential hazard, *n*_obs_ = 1000 DGM for all models, including a 95th percentile knot. Estimated hazards underlying these HRs are included in Appendix S4, and the equivalent plot but for models excluding the 95th percentile knot is included in Appendix S5.

Given sufficient spline flexibility, estimated HRs accurately capture the shape of true 3‐year HR. With earlier constraints or fewer df over the 3 years of follow‐up, HRs can be seen to underestimate truths at earlier times and overestimate truths at later ones. Mean ∆RMST percent bias was no more than 1.1% over this time for any panels in Figure 5. The majority of observed under/overestimation in hazards is integrated out in the calculation of survival estimates.

With an earlier constraint, estimated HRs approach 1 faster. With a 5‐year constraint, extrapolated HRs increase to 1 rapidly, often starting during the observed period. With later constraints, the rate of effect loss slows. The 20‐year constraint constitutes the least conservative assumption, while placing the upper boundary knot at 5 years is clearly the most conservative. Even with a constraint at 20 years, the extrapolated HR is roughly 1 by 15 years; however.

With no constraints, hazards over the first 3 years are very close to true values. Extrapolations can be seen to perform poorly; however, this is greatly worsened by the inclusion of the 95th percentile, something that would not be standard practice when extrapolating splines without constraints.

Considering the *n*_obs_ = 600 case, mean estimates were similar, but with an increased spread in individual simulation estimates. The same was true when considering the comparable low hazard scenario. These plots are both included in Appendix S6.

To investigate how the varying HR structures impact extrapolated survival estimates, 40‐year absolute marginal (∆) RMSTs are considered for the high exponential hazard, *n*_obs_ = 1000 DGM, for 5 degrees of freedom, plus a 95th percentile knot, cases (corresponding to the bottom row of models in Figure 5, see Appendix S4 for their corresponding hazards). Mean placebo/treatment (∆) RMST for the 5‐year constraint was 3.72/4.99 (1.27). For a constraint at 10 years, this was 3.73/5.19 (1.46), corresponding to an increase in the estimated survival difference of 0.19 years. The least conservative, 20‐year constraint model led to mean marginal RMSTs of 3.73/5.38 (1.65), a further increase of 0.19 years in survival benefit. With later constraints, ∆RMSTs are less conservative; in this case, increasing by an average of 14.0% with each 5 years later the upper boundary knot was placed.

Of all 172,800 models presented above for both aims, 257 (0.15%) failed to converge. All of those that did not converge included a 95th percentile knot with the earliest (5 year) constraint and on either 5 or 6 df. None of these were the 3‐year censored models, however, and only 14 occurred in a DGM that did not incorporate instant waning at 3 years.

The further consideration of biases under calendar censoring is included in Appendix S7. Patterns in bias observed are very similar to those under data generating mechanisms without calendar censoring. Absolute biases are inflated slightly under a reduced effective sample size.

## 4 Discussion

The presented method to implement smooth HR effect waning has been shown to accurately estimate RMST with full waning follow‐up and over a short‐term follow‐up, with waning extrapolated given fair modeling assumptions. Placing the upper boundary knot further from the end of follow‐up leads to less conservative estimates, while an earlier constraint leads to more conservative estimates. This provides a method to wane *conditional* HRs, important for accurate estimates, in a model from which predictions can be derived to easily calculate marginal survival time difference measures for *population‐level* decision making in HTA, in a unified framework, all while not requiring a PH assumption.

Significant bias was introduced in 20‐year censored models when constraints were placed prior to true waning. In real‐life scenarios, however, these constraints would be used to assess sensitivity to waning over extrapolated periods, given that there is no evidence for or against. While the most stringent assumptions may induce bias in extrapolated estimates, with no observed data to fit to, assumptions that are too relaxed will also bias estimates. A range of assumptions are commonly employed to evaluate the impact of plausible waning.

There was also bias introduced in several cases where constraints were placed at the earliest time point of 5 years, both in models of fully observed data and in 3‐year RMST for extrapolated waning models. Most commonly, these were models with relatively few degrees of freedom for treatment effect splines, indicating insufficient flexibility for splines to fit to observed data prior to waning *and* the waning constraints. This was minimized with the introduction of extra degrees of freedom, most effectively by placing a knot at the 95th percentile of data. Generally, a treatment effect spline in a model employing waning constraints may require an increase in degrees of freedom generally assigned to such a spline in practice.

Other factors influencing bias that are largely out of an analyst's control are the overall hazard rate and sample size. It is no surprise that increasing the sample size increases precision in estimates, while a higher hazard rate implies more events and a higher *effective* sample size.

Non‐convergence was generally very low (0.15%). All non‐convergence was observed in cases with waning fully observed, which is not reflective of a real‐life scenario. These were also concentrated around the instant waning scenario, perhaps a less clinically plausible case. Models that did not converge tended to have more knots, so if convergence issues are encountered in real‐life scenarios, it may be favorable to reduce model complexity. The convergence rate in R analyses was poorer without allowing a high number of iterations (2000), this could be attributable to inefficiencies in the altered maximization algorithm defined.

The inclusion of calendar censoring showed no evidence of any systematic bias, with patterns in bias very similar to those discussed in the scenarios without. The small inflation in absolute biases is likely attributable to a reduction in effective sample size, due to the increased rate of censoring, and hence fewer events included in the analysis.

An example of use has been provided along with an extension allowing PH over the trial follow‐up, followed by smooth waning. A further example for use may be when a decreasing or attenuating damaging effect of a prognostic factor is extrapolated. In this case, long‐term estimates of effect may indicate a reverse in direction or a protective effect, even if this is not deemed clinically plausible. For instance, it may be believed that socio‐economic status is unlikely to ever provide a protective effect; however, if the HR comparing high to low deprivation groups over an observed period is decreasing (or approaching 1), extrapolation could lead to an implied protective effect into the long‐term follow‐up. To constrain this extrapolation to a more feasible value, it may be fixed at 1 from a given time, stopping the HR from going below 1. Alternatively, this could be constrained to a constant value that could be decided or estimated based on the data, which would then be assumed into long‐term follow‐up. A perhaps more complicated example/extension of this method might be to derive more accurate long‐term RCT estimates using disease registries. Baseline comparator hazard spline coefficients might be derived based on such registry data, and a waning treatment effect estimated based on an RCT cohort. Assuming HR transportability, these could then be used to accurately estimate long‐term survival of an untreated population and get an estimate of what their survival might have been had they received treatment, better accounting for elements such as other‐cause or background mortality that may not be extrapolated based on a short‐term RCT follow‐up. Other applications exist in the relative survival framework [33]. Without assessment in simulation, further work is required to assess the properties of these approaches before recommendations can be made for their use. Further, clinical appropriateness should drive the use of these constraints in practice.

Limitations of this approach include the relative complexity of a spline‐based model in comparison to a more standard parametric PH model (e.g., exponential or Weibull baseline hazard models) and the added requirement to define the number/placement of knots over the whole follow‐up. Further, the identified potential for changes in covariate effect estimates with/without waning assumptions could require modeling of treatment‐covariate interactions if this is expected to be a problem. Commonly in an RCT, however, the exact coefficient of other covariates is not of interest, but rather the added precision their inclusion can bring or the limitation of bias in the treatment effect waning case.

The heterogeneity term, z, was included in DGMs and models before being marginalized over to derive marginal RMSTs due to previous work showing that conditioning is important in waning analyses; regression standardization was hence an important step to include. It was deemed important that observations made about hazard/HR estimates were fully attributable to modeling choices made rather than changing prognostic factor distributions over time; however, the ability to adjust for all heterogeneity is unrealistic. Implications with unmodeled heterogeneity are important to consider.

As further work, analytical SEs might be considered using techniques such as estimating equation methods (M‐estimation) [34], to reduce the computational burden required by a bootstrap process. Convergence issues in the R code used might also be alleviated by using a more efficient constrained optimization procedure. Other extensions include the assessment of later percentiles than the 95th for extra knots, the consideration of bounded constraints (to ensure HR extrapolations > 1 for example) or exploring techniques to minimize differences in HR estimates at early time points induced by constraints effective from later time points, such as adding *multiple* knots at later times or constraints on early treatment effect spline variables to their unconstrained values (provided the intercept is dealt with appropriately). This work is limited to Phase II methodological research [32] and hence further evaluation of the method in increasingly complex scenarios may be required.

We believe this method has the potential to supplement any HTA submissions where the consideration of sensitivity of conclusions to treatment effect waning is deemed valuable. Given the research question is almost always to be the consideration of treatment effect waning at the individual level, it is important that an HR waned under this framework is adjusted for all reasonable prognostic factors. Slightly more degrees of freedom will likely be required for the treatment effect spline function such that the full, probably non‐PH [7], un‐waned effect can be considered over the observed period whilst allowing sufficient flexibility to not be unduly affected by waning constraints beyond follow‐up; we recommend implementing this by including a knot at the 95th percentile of event times. If the model fails to converge, the degrees of freedom could be decreased. Regression standardization should be used to regain marginal survival estimates from a conditional model. Given that there will still likely be unadjusted heterogeneity in the model, it may also be valuable to constrain the HR to a range of effects that are small in magnitude but harmful in direction, to assess the sensitivity of results to unadjusted frailty [8]. If sensitivity to the assumption of complete waning is of interest, a range of effects that are small in magnitude but protective in direction might also be considered. This is the only way to consider uncertainty in the HR itself from the point of waning as, by the nature of the analyst‐specified constraint, there is no uncertainty accounted for in the model. Similarly, a range of timepoints from which the treatment effect is lost might be considered. As with all FPMs, the modeled hazards and HR should be considered graphically to support model assessment.

### 4.1 Conclusion

A method has been described employing flexible manipulation of splines to achieve smooth conditional HR waning, in a modeling framework that facilitates simple predictions and hence use of regression standardization to return to marginal survival estimates. Performance given fully observed waning has been demonstrated as well as the more realistic case of waning in extrapolated periods, demonstrating how a more or less conservative survival estimate might be achieved. Generally, increased degrees of freedom for treatment splines (especially including knots artificially placed at later follow‐up times) are important for accurate estimates. Treatment effect waning assumptions require justification in the face of little supporting data, and this method provides an alternative way to define these assumptions.

## Conflicts of Interest

Mr. Angus C. Jennings, Dr. Mark J. Rutherford, and Prof. Paul C. Lambert report grants from the National Institute of Health Research, during the conduct of the study. Dr. Mark J. Rutherford reports personal fees from the Association of the British Pharmaceutical Industry, outside the submitted work.

## Supporting information


**Data S1.** Supporting Information.

**Data S2.** Supporting Information.

**Data S3.** Supporting Information.

**Data S4.** Supporting Information.

**Data S5.** Supporting Information.

## Data Availability

The data that support the findings of this study are available from the corresponding author upon reasonable request.

## References

[1] National Institute for Health and Care Excellence , “NICE Health Technology Evaluations: The Manual” (2022), https://www.nice.org.uk/process/pmg36/chapter/introduction‐to‐health‐technology‐evaluation.

[2] T. Kongnakorn , G. Sarri , A. Freitag , et al., “Modeling Challenges in Cost‐Effectiveness Analysis of First‐Line Immuno‐Oncology Therapies in Non‐small Cell Lung Cancer: A Systematic Literature Review,” PharmacoEconomics 40 (2022): 183–201. 34595671 10.1007/s40273-021-01089-4 PMC8795065

[3] X. Armoiry , X. Wang‐Steverding , M. Connock , et al., “Is the Assumption of Waning of Treatment Effect Applied Consistently Across NICE Technology Appraisals? A Case‐Study Focusing on Disease‐Modifying Therapies for Treatment of Multiple Sclerosis,” International Journal of Technology Assessment in Health Care 38, no. 1 (2022): e83. 36510406 10.1017/S0266462322003269

[4] L. A. Trigg , G. Melendez‐Torres , A. Abdelsabour , and D. Lee , “Treatment Effect Waning Assumptions: A Review of NICE Technology Appraisals,” Value in Health 27 (2024): 1003–1011. 38679289 10.1016/j.jval.2024.04.016

[5] B. S. Woods , E. Sideris , S. Palmer , N. Latimer , and M. Soares , “Partitioned Survival and State Transition Models for Healthcare Decision Making in Oncology: Where Are We Now?,” Value in Health 23, no. 12 (2020): 1613–1621. 33248517 10.1016/j.jval.2020.08.2094

[6] F. Kamgar , S. Ho , E. Hawe , and T. Brodtkorb , “EE228 A Review of Treatment Effect Waning Methods for Immuno‐Oncology Therapies in National Institute for Health and Care Excellence Technology Appraisals,” Value in Health 25 (2022): S98.

[7] M. J. Stensrud and M. A. Hernán , “Why Test for Proportional Hazards?,” Journal of the American Medical Association 323, no. 14 (2020): 1401–1402. 32167523 10.1001/jama.2020.1267 PMC11983487

[8] A. C. Jennings , M. J. Rutherford , N. R. Latimer , M. J. Sweeting , and P. C. Lambert , “Perils of Randomized Controlled Trial Survival Extrapolation Assuming Treatment Effect Waning: Why the Distinction Between Marginal and Conditional Estimates Matters,” Value in Health 27, no. 3 (2024): 347–355. 38154594 10.1016/j.jval.2023.12.008

[9] P. Royston and M. K. Parmar , “Flexible Parametric Proportional‐Hazards and Proportional‐Odds Models for Censored Survival Data, With Application to Prognostic Modelling and Estimation of Treatment Effects,” Statistics in Medicine 21, no. 15 (2002): 2175–2197. 12210632 10.1002/sim.1203

[10] M. J. Rutherford , P. C. Lambert , and M. J. Sweeting , “NICE DSU TECHNICAL SUPPORT DOCUMENT 21: Flexible Methods for Survival Analysis” (2020).

[11] R Core Team , R: A Language and Environment for Statistical Computing (R Foundation for Statistical Computing, 2021).

[12] StataCorp , Stata Statistical Software: Release 18 (StataCorp LLC, 2023).

[13] M. J. Crowther and P. C. Lambert , “A General Framework for Parametric Survival Analysis,” Statistics in Medicine 33, no. 30 (2014): 5280–5297. 25220693 10.1002/sim.6300

[14] T. P. Morris , A. S. Walker , E. J. Williamson , and I. R. White , “Planning a Method for Covariate Adjustment in Individually Randomised Trials: A Practical Guide,” Trials 23, no. 1 (2022): 328. 35436970 10.1186/s13063-022-06097-z PMC9014627

[15] R. Daniel , J. Zhang , and D. Farewell , “Making Apples From Oranges: Comparing Noncollapsible Effect Estimators and Their Standard Errors After Adjustment for Different Covariate Sets,” Biometrical Journal 63, no. 3 (2021): 528–557. 33314251 10.1002/bimj.201900297 PMC7986756

[16] C. De Boor , A Practical Guide to Splines (Springer‐Verlag google schola, 1978).

[17] W. Wang and J. Yan , “Shape‐Restricted Regression Splines With R Package splines2,” Journal of Data Science 19, no. 3 (2021): 498–517, 10.6339/21-JDS1020.

[18] T. M. Andersson , P. W. Dickman , S. Eloranta , and P. C. Lambert , “Estimating and Modelling Cure in Population‐Based Cancer Studies Within the Framework of Flexible Parametric Survival Models,” BMC Medical Research Methodology 11, no. 1 (2011): 1–11. 21696598 10.1186/1471-2288-11-96 PMC3145604

[19] P. Lambert , “STPM3: Stata Module to Fit Flexible Parametric Survival Models” (2023).

[20] B. Efron and R. J. Tibshirani , An Introduction to the Bootstrap (CRC Press, 1994).

[21] M. J. Rutherford , M. J. Crowther , and P. C. Lambert , “The Use of Restricted Cubic Splines to Approximate Complex Hazard Functions in the Analysis of Time‐To‐Event Data: A Simulation Study,” Journal of Statistical Computation and Simulation 85, no. 4 (2015): 777–793.

[22] J. A. Laurie , C. G. Moertel , T. R. Fleming , et al., “Surgical Adjuvant Therapy of Large‐Bowel Carcinoma: An Evaluation of Levamisole and the Combination of Levamisole and Fluorouracil. The North Central Cancer Treatment Group and the Mayo Clinic,” Journal of Clinical Oncology 7, no. 10 (1989): 1447–1456. 2778478 10.1200/JCO.1989.7.10.1447

[23] M. Fauvernier , L. Remontet , Z. Uhry , N. Bossard , and L. Roche , “survPen: An R Package for Hazard and Excess Hazard Modelling With Multidimensional Penalized Splines,” Journal of Open Source Software 4, no. 40 (2019): 1434.

[24] C. H. Jackson , “Flexsurv: A Platform for Parametric Survival Modeling in R,” Journal of Statistical Software 70 (2016): 1–33. 10.18637/jss.v070.i08 PMC5868723 29593450

[25] T. P. Morris , I. R. White , and M. J. Crowther , “Using Simulation Studies to Evaluate Statistical Methods,” Statistics in Medicine 38, no. 11 (2019): 2074–2102. 30652356 10.1002/sim.8086 PMC6492164

[26] R. Bender , T. Augustin , and M. Blettner , “Generating Survival Times to Simulate Cox Proportional Hazards Models,” Statistics in Medicine 24, no. 11 (2005): 1713–1723. 15724232 10.1002/sim.2059

[27] M. J. Crowther and P. C. Lambert , “Simulating Biologically Plausible Complex Survival Data,” Statistics in Medicine 32, no. 23 (2013): 4118–4134. 23613458 10.1002/sim.5823

[28] J. Peron , G. R. Pond , H. K. Gan , et al., “Quality of Reporting of Modern Randomized Controlled Trials in Medical Oncology: A Systematic Review,” Journal of the National Cancer Institute 104, no. 13 (2012): 982–989. 22761273 10.1093/jnci/djs259

[29] National Institute for Health and Care Excellence , “Darolutamide With Androgen Deprivation Therapy and Docetaxel for Treating Hormone‐Sensitive Metastatic Prostate Cancer” (2023). 40146870

[30] S. L. Brilleman , R. Wolfe , M. Moreno‐Betancur , and M. J. Crowther , “Simulating Survival Data Using the Simsurv R Package,” Journal of Statistical Software 97 (2021): 1–27.

[31] E. L. Kaplan and P. Meier , “Nonparametric Estimation From Incomplete Observations,” Journal of the American Statistical Association 53, no. 282 (1958): 457–481.

[32] G. Heinze , A. L. Boulesteix , M. Kammer , T. P. Morris , I. R. White , and Initiative SPotS , “Phases of Methodological Research in Biostatistics—Building the Evidence Base for New Methods,” Biometrical Journal 66, no. 1 (2024): 2200222. 10.1002/bimj.202200222 PMC7615508 36737675

[33] M. J. Sweeting , M. J. Rutherford , D. Jackson , et al., “Survival Extrapolation Incorporating General Population Mortality Using Excess Hazard and Cure Models: A Tutorial,” Medical Decision Making 43, no. 6 (2023): 737–748. 37448102 10.1177/0272989X231184247 PMC10422853

[34] L. A. Stefanski and D. D. Boos , “The Calculus of M‐Estimation,” American Statistician 56, no. 1 (2002): 29–38.

