# Supplementary material for: A Spline‐Based Approach to Smoothly Constrain Hazard Ratios With a View to Apply Treatment Effect Waning
Source: Stat Med. 2025 Mar 9;44(6):e70035. doi: 10.1002/sim.70035 (PMC11891414; doi:10.1002/sim.70035)
Supplement: Supplementary file 1 — Data S1. Supporting Information. [file SIM-44-0-s005.docx]

**Appendix**

***Appendix 1***

*Conditional (on mean/mode covariate values) hazard, survival probability and HR estimates and 95% confidence intervals from PH models, without and with waning assumptions, with rug plot of event times.*

**
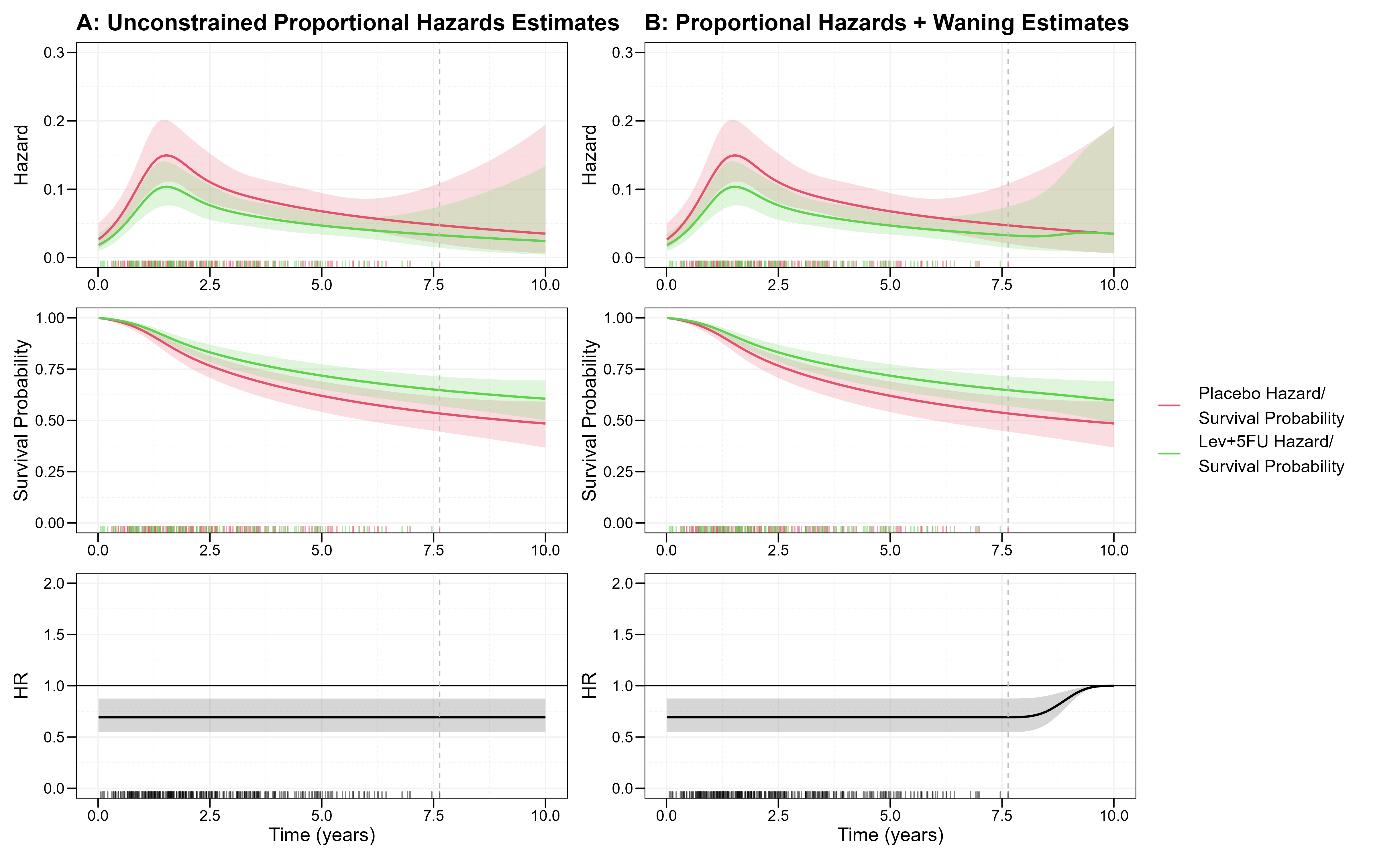
**

*Vertical dashed line indicates last event time and the start of waning for model B.*

***Appendix 2***

*Mean absolute bias in 20-year ∆RMST for all data-generating mechanisms.*


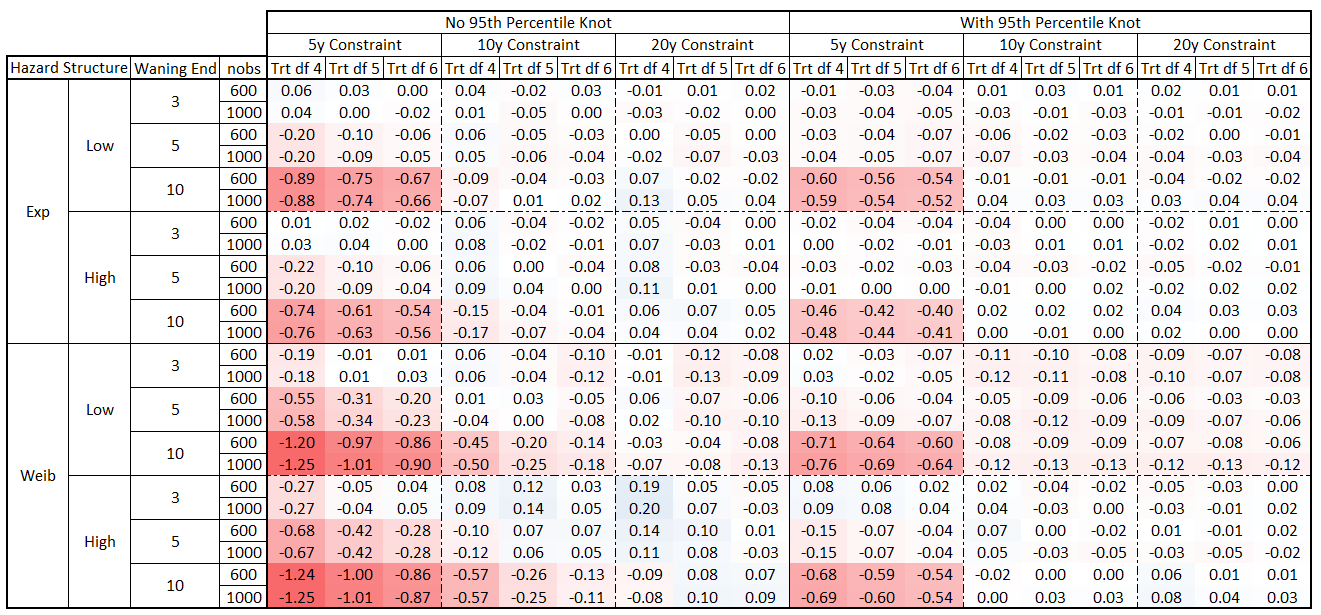


*df – degrees of freedom (here of the time-varying treatment effect hazard ratio); Exp – Exponential; Trt – treatment; Weib - Weibull*

***Appendix 3***

*Mean absolute bias in 3-year ∆RMST for data generating mechanisms with waning end at 3 years.*


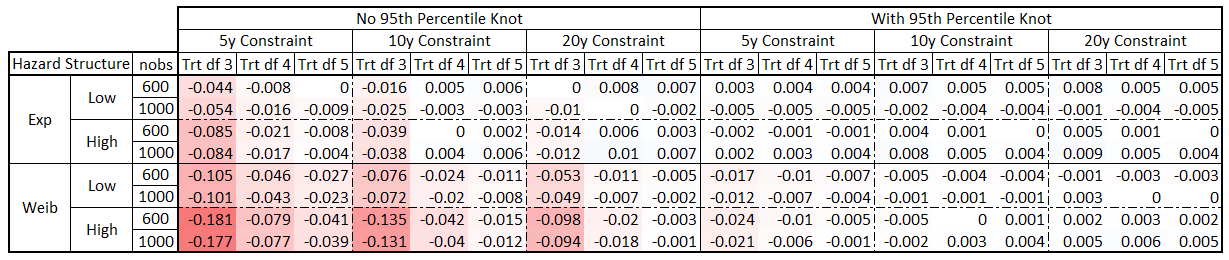


*df – degrees of freedom (here of the time-varying treatment effect hazard ratio); Exp – Exponential; Trt – treatment; Weib - Weibull*

***Appendix 4***

*Conditional (on z=0)* ***40-year hazards*** *for the first 100 of 300 simulations under the high exponential hazard data generating mechanism, sized 1000 each, all models including a 95^th^ percentile knot.*


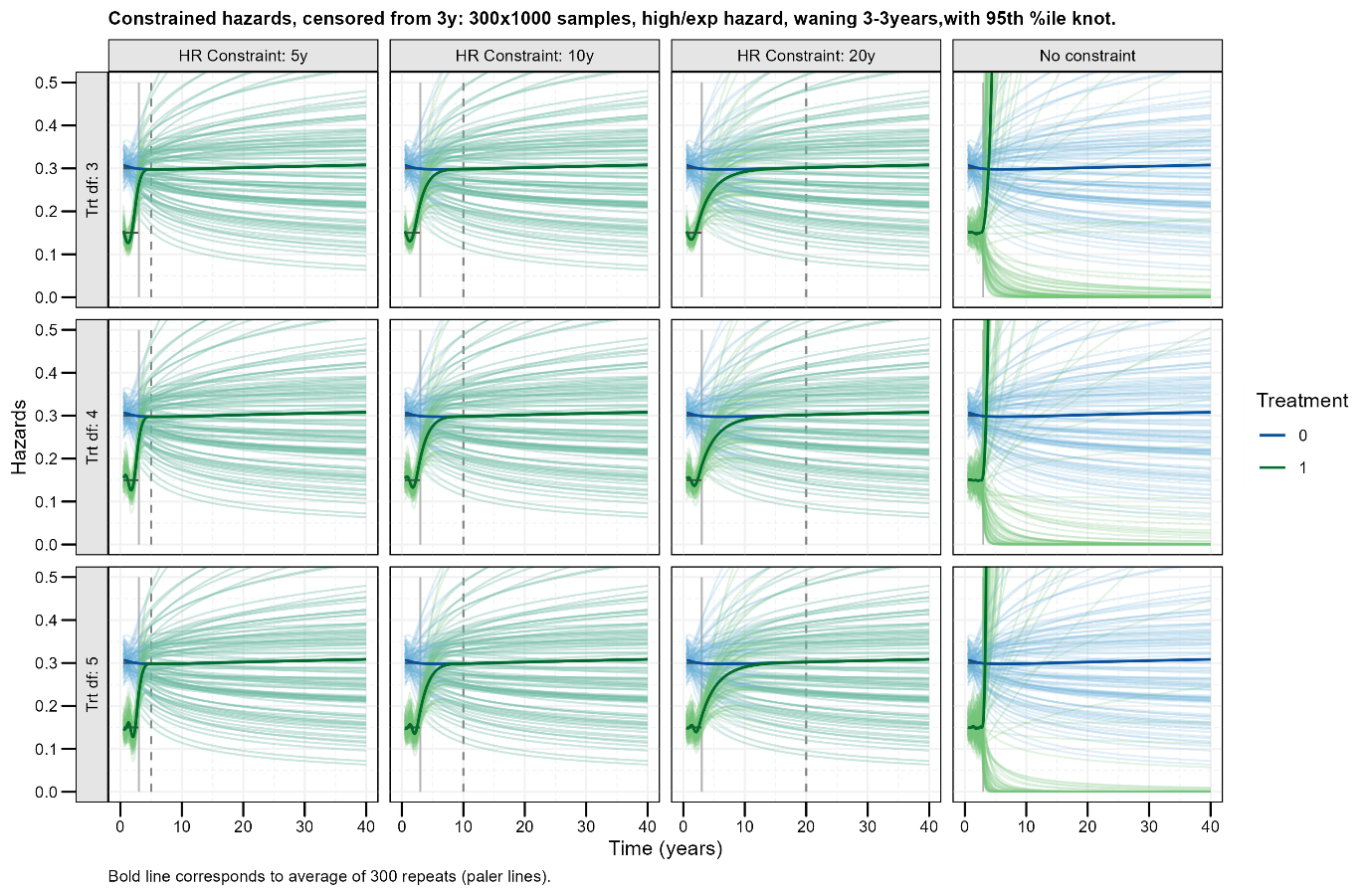


*Darker line corresponds to the average of the 100 simulations (paler lines). The solid vertical line at 3 years indicates point of censoring. The true conditional HR of 0.5 is indicated in grey prior to this point. Dashed vertical lines at 5, 10 and 20 years indicate the point of constraint*

***Appendix 5***

*Conditional (on z=0) hazard ratios for the first 100 of 300 simulations under the high exponential hazard data generating mechanism, sized 1000 each, all models* ***excluding*** *a 95^th^ percentile knot.*


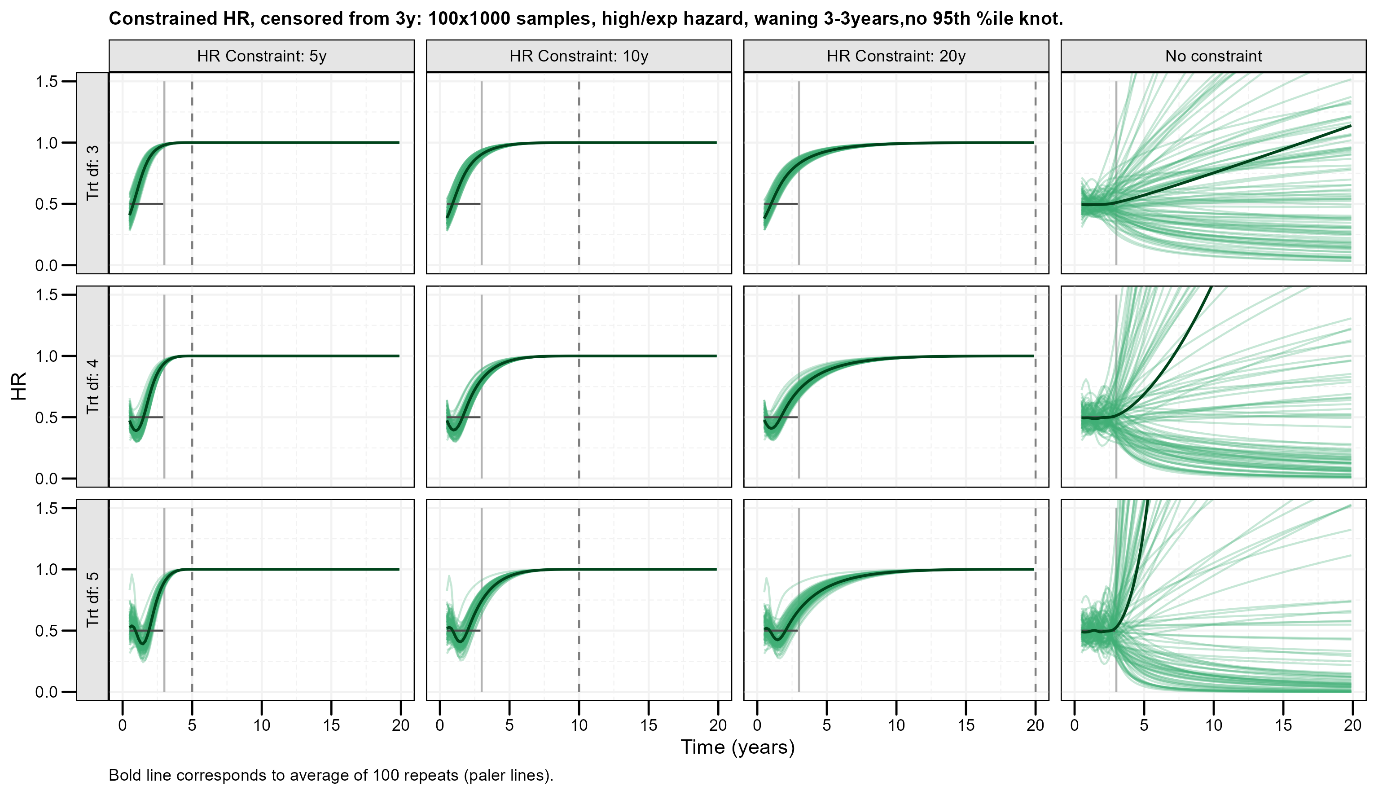


*Darker line corresponds to the average of the 100 simulations (paler lines). The solid vertical line at 3 years indicates point of censoring. The true conditional HR of 0.5 is indicated in grey prior to this point. Dashed vertical lines at 5, 10 and 20 years indicate the point of constraint*

***Appendix 6***

Low Hazard Models

*Conditional (on z=0) hazard ratios for the first 100 of 300 simulations under the* ***low exponential hazard*** *data generating mechanism, sized 1000 each, all models including a 95^th^ percentile knot.*

***
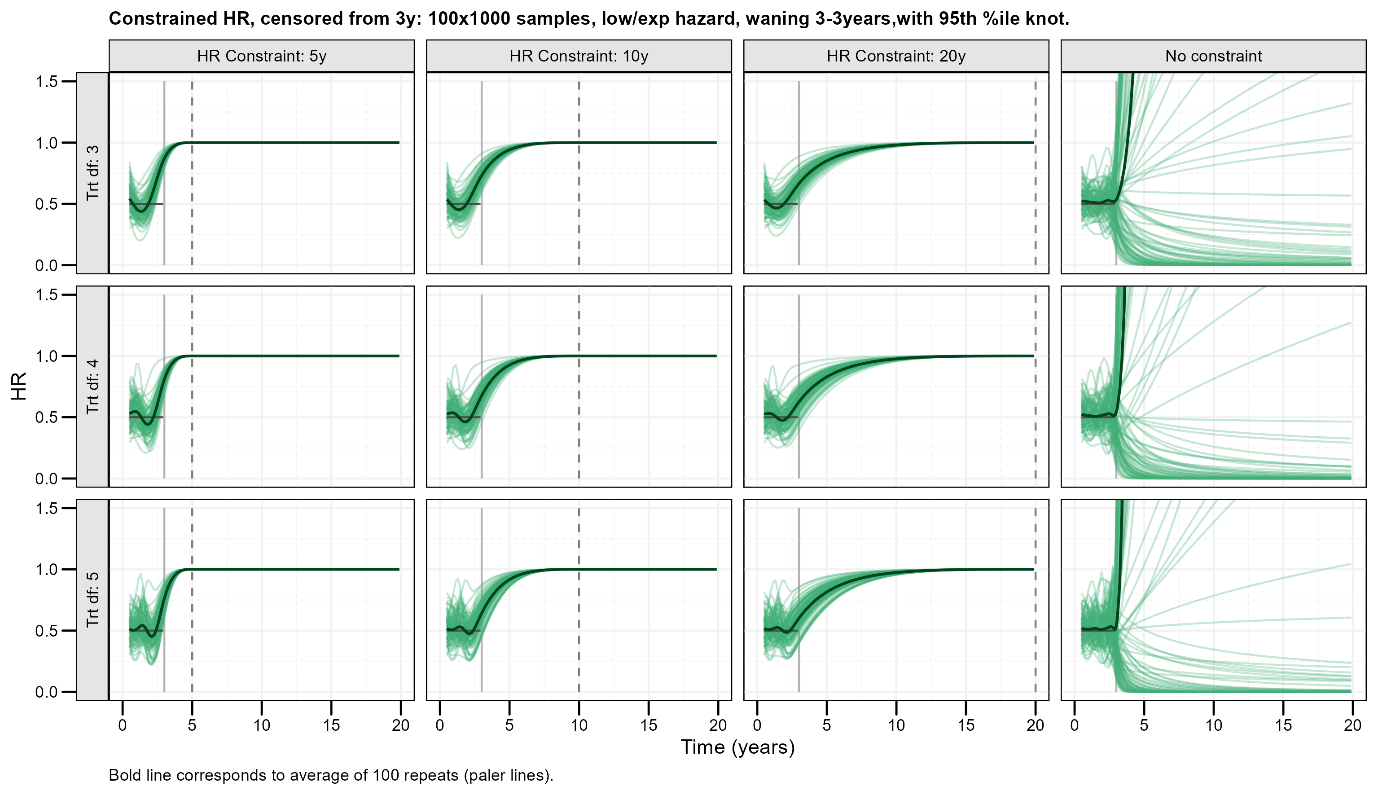
***

*Darker line corresponds to the average of the 100 simulations (paler lines). The solid vertical line at 3 years indicates point of censoring. The true conditional HR of 0.5 is indicated in grey prior to this point. Dashed vertical lines at 5, 10 and 20 years indicate the point of constraint*

Smaller sample size models

*Conditional (on z=0) hazard ratios for the first 100 of 300 simulations under the high exponential hazard data generating mechanism,* ***sized 600 each****, all models including a 95^th^ percentile knot.*

***
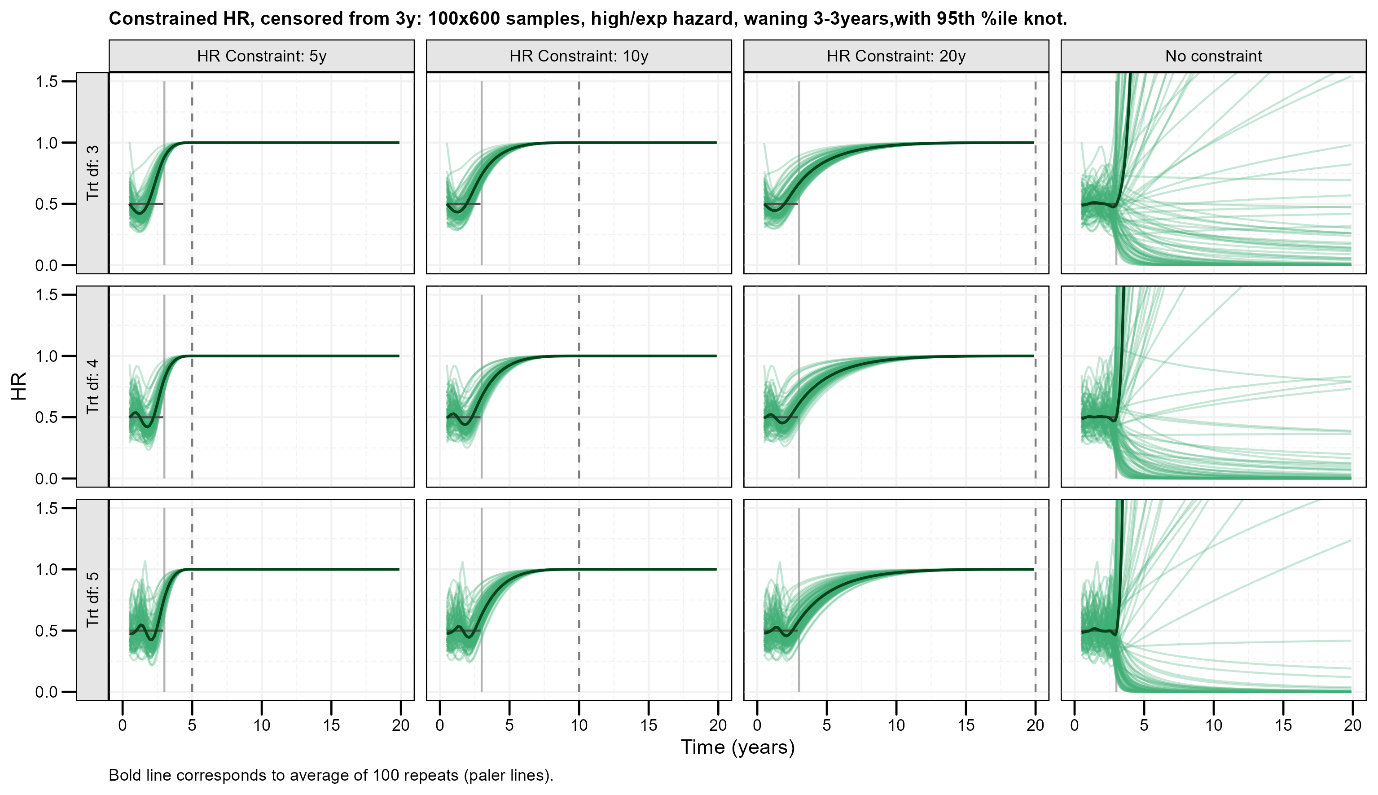
***

*Darker line corresponds to the average of the 100 simulations (paler lines). The solid vertical line at 3 years indicates point of censoring. The true conditional HR of 0.5 is indicated in grey prior to this point. Dashed vertical lines at 5, 10 and 20 years indicate the point of constraint*

***Appendix 7***

*Mean percent bias in 3-year ∆RMST for data generating mechanisms with waning end at 3 years; calendar censoring.*


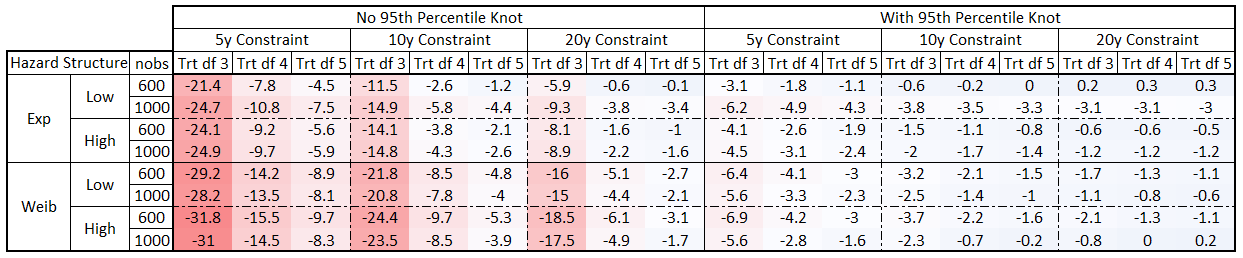


*df – degrees of freedom (here of the time-varying treatment effect hazard ratio); Exp – Exponential; Trt – treatment; Weib - Weibull*
